# qCon: QoS-Aware Network Resource Management for Fog Computing

**DOI:** 10.3390/s18103444

**Published:** 2018-10-13

**Authors:** Cheol-Ho Hong, Kyungwoon Lee, Minkoo Kang, Chuck Yoo

**Affiliations:** 1 School of Electrical and Electronics Engineering, Chung-Ang University, 84 Heukseok-ro, Dongjak-gu, Seoul 06974, Korea; cheolhohong@cau.ac.kr; 2 Department of Computer Science and Engineering, Korea University, 145 Anam-ro, Seongbuk-gu, Seoul 02841, Korea; kwlee@os.korea.ac.kr (K.L.); kangsdom@korea.ac.kr (M.K.)

**Keywords:** fog computing, IoT architecture, QoS policy, network resource management

## Abstract

Fog computing is a new computing paradigm that employs computation and network resources at the edge of a network to build small clouds, which perform as small data centers. In fog computing, lightweight virtualization (e.g., containers) has been widely used to achieve low overhead for performance-limited fog devices such as WiFi access points (APs) and set-top boxes. Unfortunately, containers have a weakness in the control of network bandwidth for outbound traffic, which poses a challenge to fog computing. Existing solutions for containers fail to achieve desirable network bandwidth control, which causes bandwidth-sensitive applications to suffer unacceptable network performance. In this paper, we propose qCon, which is a QoS-aware network resource management framework for containers to limit the rate of outbound traffic in fog computing. qCon aims to provide both proportional share scheduling and bandwidth shaping to satisfy various performance demands from containers while implementing a lightweight framework. For this purpose, qCon supports the following three scheduling policies that can be applied to containers simultaneously: proportional share scheduling, minimum bandwidth reservation, and maximum bandwidth limitation. For a lightweight implementation, qCon develops its own scheduling framework on the Linux bridge by interposing qCon’s scheduling interface on the frame processing function of the bridge. To show qCon’s effectiveness in a real fog computing environment, we implement qCon in a Docker container infrastructure on a performance-limited fog device—a Raspberry Pi 3 Model B board.

## 1. Introduction

Centralized cloud computing platforms such as Amazon EC2 and the Google Cloud Platform have become a prevalent approach to collect and process massive Internet of Things (IoT) data generated by countless sensors, micro-cameras, and smart-objects; in the literature, when a cloud infrastructure is constructed on the core of the network, the cloud is regarded as centralized [1,2]. IoT application developers are increasingly moving their applications to cloud services, as they are always available, robust, and reliable [3]. Nevertheless, centralized clouds require IoT developers to deploy their applications into a geographically distant data center. As the computation and storage resources are remote from IoT devices, end users suffer low bandwidth, high network latency, and deficient responsiveness. This limitation eventually leads to a poor experience for end users utilizing traditional cloud services.

Recent fog computing architectures overcome the limitation of traditional clouds by placing computation resources near IoT devices [4,5]. Fog computing exploits computation and network resources at the edge of a network to build small clouds, which perform as small data centers. Fog computing employs network gateways, routers, and WiFi access points (APs), which construct data paths between IoT devices and internet service providers, as a distributed cloud computing platform. A great deal of data produced by IoT devices can be collected and analyzed in these computation resources closer to IoT devices, which allows higher bandwidth, lower latency, improved responsiveness, and a better user experience. Besides fog computing, there are several computing paradigms that utilize computation resources at the edge. For example, edge computing employs computation and network resources at the edge of a network without building a cloud [1]. Osmotic computing constructs a federated computing environment between a data center at the edge and a data center at the traditional cloud by enabling automatic deployment of micro services that are interconnected over both infrastructures [6].

In recent years, containers [7] have been used as a fog computing infrastructure because they enable lightweight virtualization for performance-limited edge devices such as WiFi APs and set-top boxes employed as network gateways [8,9]. As containers are multiplexed by a single operating system (OS) kernel, they do not require an additional software layer for virtualization (e.g., hypervisors) compared to virtual machine technologies. This feature allows containers to begin and finish quickly and to achieve near-native performance [7]. Recent edge devices show a tendency to be equipped with single-board computers to offer better power efficiency. Leveraging containers in a fog computing platform is obviously an attractive choice for such low-power devices.

Unfortunately, containers have a weakness in the control of network bandwidth for outbound traffic, which poses a challenge to fog computing. Recent container engines such as Docker [10] do not provide their own quality of service (QoS) mechanism for controlling bandwidth. They send traffic in a best-effort manner, and therefore bandwidth-intensive or real-time multimedia applications would suffer unacceptable network performance. To address this issue, they suggest exploiting Linux Traffic Control [11] to configure each container’s network performance for outbound traffic. However, the existing scheduling policies of Linux Traffic Control provide limited and incomplete functionalities [12], which cannot ensure efficient control of network bandwidth on containers in fog computing. Linux Traffic Control has limitations in that (1) it cannot guarantee proportional share bandwidth distribution as specified in each container’s parameters on a performance-limited device, which will be explained in Section 2.3, (2) it cannot simultaneously apply both proportional share scheduling and bandwidth shaping to containers [11], and finally (3) it incurs high CPU overhead [13].

In this paper, we propose qCon, which is a QoS-aware network resource management framework for containers in fog computing. QoS is an important metric for fog applications and can be classified into four categories as follows: connectivity, reliability, capacity (or network bandwidth), and delay [14]. The main problem that this article tries to address belongs to the capacity category among the four items. qCon is able to differentiate each container’s outbound network performance according to the container’s characteristic or the price paid for network resources. qCon’s objective is to efficiently limit the outbound rate of TCP and UDP traffic to satisfy different network performance requirements, which are described in terms of network bandwidth. For this purpose, qCon supports three scheduling policies that can be applied to containers simultaneously: proportional share scheduling, minimum bandwidth reservation, and maximum bandwidth limitation. Proportional share scheduling offers relative performance based on the weight of each container [15]. Another objective of qCon is to implement a lightweight framework to be used in containers for performance-limited fog devices such as WiFi APs and set-top boxes. For this purpose, qCon is embedded in the Linux bridge, which connects the host operating system network and the internal network for containers, and conducts network scheduling during packet processing of the Linux bridge in a synchronized manner.

**Figure 1:**
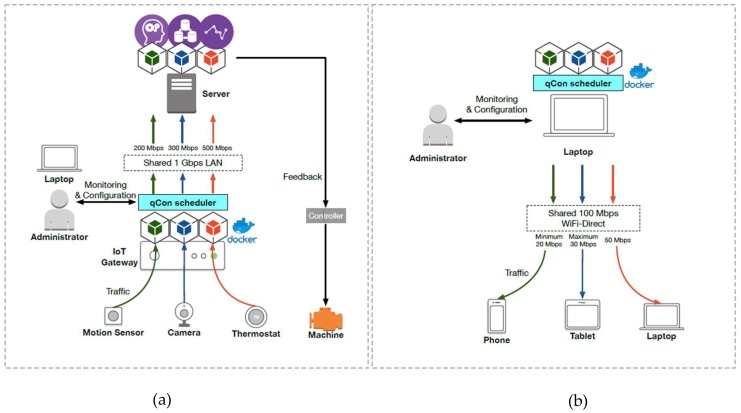
Use cases of qCon (**a**) in a smart factory and (**b**) in fog computing using mobile devices. IoT: Internet of Things; LAN: local area network.

Figure 1 suggests two use cases of qCon. The left figure shows a smart factory where an edge resource (i.e., IoT gateway) receives raw data from sensors, preprocesses and filters the received data, and sends the filtered data to the private cloud server in the same factory [16]. The private cloud can protect privacy for sensitive data and offer faster response than a public cloud. The cloud server performs comprehensive data analytics using machine learning and big data technologies and sends immediate feedback to the controller to control factory machines. The IoT gateway is equipped with containers to offer an isolated environment to each application filtering different sensor data. In this scenario, qCon is responsible for differentiating each container’s outbound network performance according to the application’s characteristic when sending traffic to the server. For example, the administrator can assign a container running a bandwidth-intensive application a high weight value for proportional share scheduling, so that the container can reliably send packets to the cloud server at a high rate.

In the right figure, qCon is used in a fog computing environment where mobile devices such as smartphones and laptops form a small cloud in a coffee shop or a university [17]. The mobile devices are directly connected with each other by WiFi-Direct for fast communication [18]. In this use case, an idle device with sufficient capability can provide its computation resources to other tenants and receive an incentive. The mobile device providing computation resources is equipped with containers to give an isolated environment to each tenant. In this use case, qCon can effectively control each container’s network send rate as follows: First, a weight value for each container is assigned by the administrator according to the price paid for network resources. Second, if a tenant requires a quantitative network resource amount such as “minimum 20 Mbps” for real-time multimedia applications, qCon can apply the minimum bandwidth reservation policy to its container. Finally, let us suppose that the tablet in the figure has used up the network quotas allowed for a day. The administrator can apply the maximum bandwidth limitation policy to its container in order to impose a limited network speed within the same day.

To show qCon’s effectiveness in a real fog computing environment, we implement qCon in a Docker container platform on a performance-limited fog device, a Raspberry Pi 3 Model B board, and present its evaluation results. Throughout the evaluation, we show that qCon provides fine-grain bandwidth control according to each container’s requirement, incurs low CPU overhead, and accurately isolates each container’s traffic.

The remainder of this paper is structured as follows: In Section 2, we explain the background of containers, their network driver models, and network bandwidth control on containers. Section 3 elaborates on the design of qCon including the scheduler, the credit allocator, and the configuration interface. Section 4 shows the performance evaluation results. Section 5 explains related work. Section 6 suggests discussion points. Finally, we present our conclusions in Section 7.

## 2. Background

Virtual machine (VM) technologies such as KVM [19] and Xen [20] have been a popular approach to achieve elasticity in a cloud platform. In recent years, lightweight virtualization (e.g., containers) is gaining significant attention in fog computing thanks to its high performance and easy deployment. In this section, we will review the use of containers in fog computing, feasible network models in containers, and network bandwidth control on containers.

### 2.1. Containers in Fog Computing

Containers have been used as a fog platform instead of VMs because containers provide low overhead for performance-limited fog devices such as network gateways, routers, and WiFi access points (APs). Container technologies utilize namespaces and cgroups provided by the Linux kernel in order to enable fault and performance isolation between multiple containers. Namespaces partition kernel resources so that one container of a certain namespace cannot access the kernel resources of containers with other namespaces. Therefore, a container’s fault would be contained within the container boundary and could not affect other containers, implementing fault isolation. Different namespaces can be used for isolating container IDs, network interfaces, interprocess communication, and mount-points. cgroups limit and account for each container’s resource usage, including the CPU, memory, and I/O devices. For example, cgroups can limit a specific container to a configured CPU amount. In addition to fault isolation enforced by namespaces, cgroups focus on performance isolation between containers by sharing and limiting available hardware resources.

### 2.2. Network Driver Models in Containers

The container engine (e.g., Docker) supports several pluggable network drivers for containers, which provide core networking functionality [21]. The provided drivers include the host, bridge, overlay, macvlan, and none drivers. In this section, we explain the two most significant ones widely utilized in containers: the host and bridge drivers.

**Figure 2:**
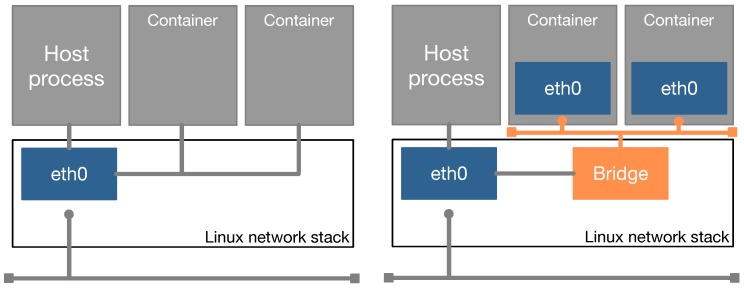
Architecture of the host driver mode (**left**) and the bridge driver mode (**right**).

The host driver allows containers to share the network established by the host, as shown on the left side of Figure 2. In this mode, packets sent to or received from containers are processed by the same network stack of the Linux kernel. The container packets are fundamentally handled in the same way in which the host processes packets. To be specific, containers in the host mode share the same media access control (MAC) and IP addresses so that packets are distributed to each container based on a port number. The host driver model is useful when multiple containers are owned by a single user and the containers communicate with each other. However, this mode does not provide an isolation feature. For example, applications having the same port number in different containers cannot send or receive packets concurrently.

The bridge driver is the default network driver configured by the container engine. In this mode, a link layer bridge of the Linux kernel is exploited. A physical bridge (or switch) essentially merges two local area networks (LANs) and makes them a unified network. Similarly, the Linux bridge configured by the container engine is a virtual network switch and connects the network of the host to the internal network established by containers, as shown on the right side of Figure 2. In bridge mode, each virtual network interface in containers (i.e., eth0 in each container) can connect to the bridge without concerning its existence. The bridge then forwards packets between the two networks based on each MAC address of the network interfaces. As each container can employ a virtual network interface inside it, the bridge mode provides an isolated network environment to each container compared with the host driver mode [22]. We select the bridge driver for our design and implementation of qCon, as this mode enables isolation between containers for multiple tenants exploiting fog computing facilities.

### 2.3. Network Bandwidth Control on Containers

**Figure 3:**
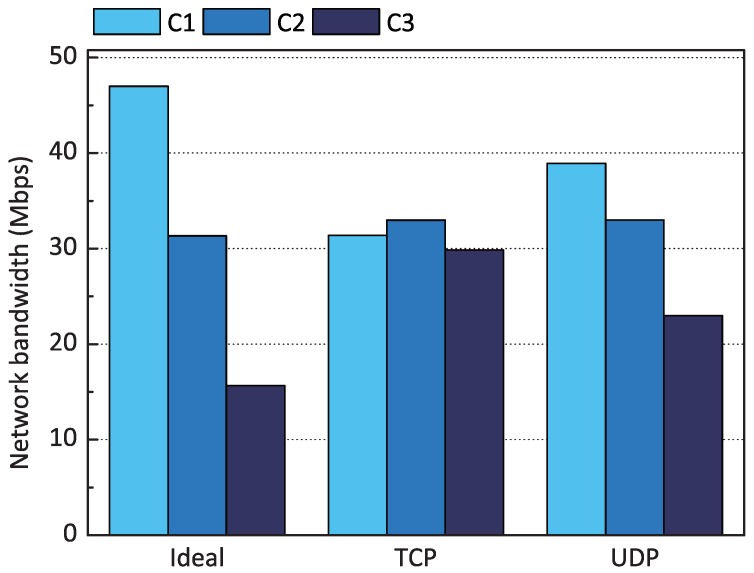
Performance of Deficit Round-Robin in Linux Traffic Control when the weights of three containers are 3:2:1, and each container sends TCP (**left**) and UDP packets (**right**).

Recent container engines suggest exploiting Linux Traffic Control [11] to adjust each container’s outbound network performance. However, we found that desirable QoS levels could not be achieved with Linux Traffic Control when we evaluated the Deficit Round-Robin (DRR) scheduler [23] in Linux Traffic Control for proportional share scheduling. Figure 3 shows the evaluation result when we assigned the weights of 3:2:1 to three containers on a fog device (i.e., Raspberry Pi). A weight reflects the container’s relative use of network resources. As shown in the figure, the DRR failed to achieve weighted fair sharing on the three containers because the scheduler efficiently works only when its scheduling queue is congested with sufficient packets to send. Unfortunately, when we sent TCP packets from the containers, we observed that each packet left the system as soon as it entered the queue. Therefore, the network performance of each of the three containers became similar, as shown in the figure. From this evaluation, we identified that it is difficult to congest a queue with sufficient packets on a performance-limited device in fog computing compared to servers in data centers. With UDP packets, the queue becomes slightly more congested, but the scheduler still cannot guarantee weighted fair sharing.

## 3. Design of qCon

In this section, we present the details of qCon, a QoS-aware network resource management framework for containers in fog computing for IoT. First, we describe the design goals of qCon that aim to provide a lightweight bandwidth control infrastructure for containers. Next, we elaborate on the qCon architecture consisting of the scheduler, the credit allocator, and the configuration interface. Finally, we explain the scheduling algorithms of qCon for achieving diverse performance goals.

### 3.1. Design Goals

The goals of qCon, which focuses on implementing a lightweight bandwidth control infrastructure for containers on fog devices, are as follows.

#### 3.1.1. Implementing a Lightweight Framework

qCon aims to provide a lightweight framework to be used in containers for performance-limited fog devices such as WiFi APs and set-top boxes. In addition to these devices, up-to-date fog computing technologies employ battery-powered and energy-limited devices such as smart phones, laptops, cars, and drones [17,24]. To exploit these devices in fog computing, qCon considers energy efficiency as an important factor and tries to achieve low CPU overhead. To satisfy this goal, qCon does not revise Linux Traffic Control, which has complicated internal implementations and incurs significant CPU overhead [25]. Instead, qCon implements its own scheduling framework on the Linux bridge by interposing qCon’s scheduling interface on a frame processing function of the bridge. qCon’s scheduling framework adopts a simple credit-based accounting mechanism so that it incurs low CPU overhead.

#### 3.1.2. Enabling a Proportional Share Scheduling Policy

Fog computing implements a decentralized cloud computing environment where tenants utilize computation resources in fog nodes close to their devices [18,26]. In a cloud computing environment, it is essential to enforce a proportional share scheduling policy for network resources on virtual entities such as containers. The proportional share scheduling policy assigns a weight value to each container, and allows the container to utilize the network resources in proportion to its weight. A weight value is assigned by the administrator according to the price paid for network resources or other conditions. The Deficit Round Robin scheduler [23] in Linux Traffic Control is supposed to enable proportional share scheduling on containers, but it fails to achieve desirable QoS levels as it works only when the scheduling queue is congested, as described in Section 2.3. This limitation prevents fog computing from giving access to network resources on a pay-per-use basis. qCon endeavors to implement a proportional share scheduling policy regardless of the congestion in the scheduling queue.

#### 3.1.3. Concurrent Support of Multiple Performance Policies

qCon aims to support multiple performance policies on each container at the same time. Recent fog computing users tend to run various network applications on their containers [27,28]. These applications obviously have different network performance requirements, which can be described in terms of network bandwidth. Minimum sustainable bandwidth can be an example for applications that process streaming videos [29]. In order to satisfy various demands of containers in fog computing, qCon offers the following three performance policies: proportional share scheduling, minimum reservation, and maximum limitation. These three policies can be simultaneously applied to a container.

### 3.2. qCon Architecture

**Figure 4:**
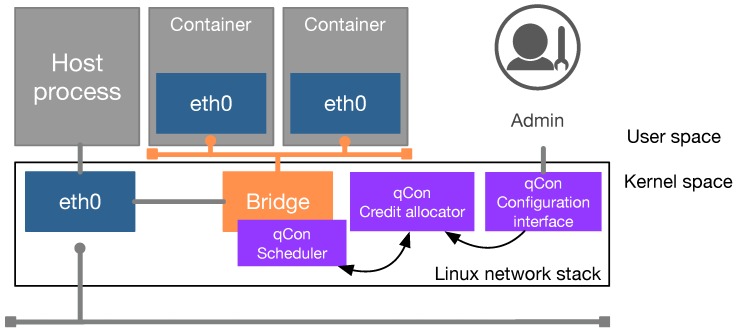
qCon components.

qCon exploits the bridge driver model for networking in order to enable isolation between containers, as explained in Section 2.2. qCon extends the Linux bridge to implement the scheduling framework and its subcomponents. As the Linux bridge is located in the kernel space of the host OS, qCon also implements its components in kernel space. Developing some components in kernel space generally requires both recompilation of the kernel source code and a reboot of the system. In order to alleviate this deployment burden, qCon’s core components are implemented as loadable kernel modules (LKMs), which can be installed without recompilation and reboot. As depicted in Figure 4, qCon consists of the following three components: the scheduler, the credit allocator, and the configuration interface.

#### 3.2.1. qCon Scheduler

**Figure 5:**
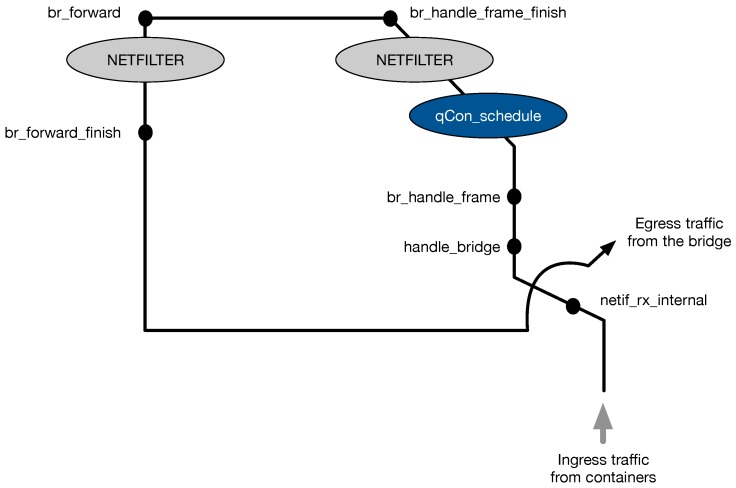
Scheduling function of qCon implemented in the Linux bridge.

The qCon scheduler is the main entity that enables qCon’s multiple performance policies, which will be explained in Section 3.3. We extend the Linux bridge to execute qCon’s scheduling function, as shown in Figure 5. When a container sends its network traffic, the *netif_rx_internal* function is executed to steer the container’s packet to the bridge. Then, the *handle_bridge* function checks whether the packet is to be sent to the bridging subsystem. If the check is successful, the *br_handle_frame* function checks whether the packet is delivered for bridging or routing. If the packet is to be bridged, the function executes a *Netfilter* callback (i.e., *NF_BR_PRE_ROUTING*) to filter or modify the packet. Before the Netfilter callback in the *br_handle_frame* function, we create a hook to call qCon’s scheduling function (i.e., *qCon_schedule*), which queues packets and selects appropriate packets for transmission according to the scheduling policies. The qCon_schedule function maintains queues for each container and processes the requests in each queue in a round-robin manner. The function selects and sends the packets of a certain queue only when its corresponding container has sufficient resource allocation. The selected packets will be consecutively processed by *br_handle_frame_finish*, *br_forward*, and *br_forward_finish*, which find the destination MAC address from the forwarding database and forward the packets to the correct bridge port.

#### 3.2.2. qCon Credit Allocator

The credit allocator periodically assigns a credit to the virtual network interface of each container based on the specified performance requirements. qCon utilizes the credit concept [30] adopted from the Xen hypervisor [20] in order to represent the amount of resource allocation. When the network resources of each container are being used, the virtual network interface of the container consumes its credit according to the requested packet sizes. The credit value of each container is periodically recharged as claimed by the weight of the container and the minimum and maximum bandwidth requirements. For this purpose, the credit allocator has a kernel timer that regularly executes a credit calculation function every 30 ms, which obtains each container’s credit value according to the performance requirements and adds the attained value to the remaining credit value. The details of scheduling mechanisms using the credit concept are given in Section 3.3.

#### 3.2.3. qCon Configuration Interface

The configuration interface allows the administrator to specify the performance requirements of each container in terms of network bandwidth. This interface utilizes the proc file system in the host OS. The proc file system reflects the current state of the OS kernel and allows the administrator to change the required state in user space. Therefore, the proc file system can be used as a communication channel between kernel and user spaces. In qCon, when a new container is created, its corresponding file is generated in the proc directory. The administrator is then able to access the created file to configure the weight, the minimum, and the maximum bandwidth of the new container. The default weight value is set to 1, and the default minimum and maximum bandwidth are set to 0, which means that the container would receive a fair share of network resources and have no lower or upper limitation of network bandwidth. The administrator can change the configured settings during runtime according to the demand of each tenant. The configuration interface also provides some useful information, such as the actual resource usage of each container during runtime.

### 3.3. Scheduling Policies

qCon provides the following three scheduling policies to satisfy various demands of containers in fog computing: proportional share scheduling, minimum bandwidth reservation, and maximum bandwidth limitation. Proportional share scheduling is the default scheduling policy in qCon, and differentiates the amount of allocated network resources proportionally based on the weight of each container. qCon also supports work-conserving [31] to maximize network resource utilization. Minimum bandwidth reservation ensures that the quantitative network resource amount of a certain container is greater than a specified amount. With this policy, qCon can support applications that require sustainable minimum bandwidth such as a multimedia applications that demand stable and quantitative network bandwidth to achieve high-quality video playback. Maximum bandwidth limitation prevents a container from consuming more than a specified amount of network resources. This policy can be used by the administrator to enforce bandwidth limitations on a container when the container has used up the quotas allowed for a day or a month. These three policies can be applied to a container simultaneously. In this case, the priority is given to maximum bandwidth limitation and minimum bandwidth reservation over proportional share scheduling.

#### 3.3.1. Proportional Share Scheduling

Proportional share scheduling is a base policy of qCon that controls a container’s network performance in proportion to its weight. The containers on the fog device are denoted by CON = {$CON_{1}$, $CON_{2},\cdots ,CON_{n}$}, where *n* is the number of containers on the fog device (*n* ≥ 1). The weight of container $CON_{i}$ is represented by $\omega (CON_{i})$, which is a relative proportion of network consumption. The sum of the weights of every container on the fog device is equal to 1. Therefore, we have $\sum_{i=1}^{n}\omega (CON_{i})=1$. Proportional share scheduling distributes network resources, which are represented as credits [30], to each container at every scheduling period (i.e., 30 ms). The credit amount for each container is calculated sequentially from $CON_{1}$ to $CON_{n}$ at the scheduling period. $C_{i}^{A}$ represents an allocated credit amount for $CON_{i}$ that can be consumed during the scheduling period. This value is the weighted fair share of network resources for $CON_{i}$ according to its weight. $C^{T}$ indicates the total amount of credits for all containers during a scheduling period. $C_{i}^{A}$ is then calculated as follows:

(1) $$\begin{array}{c} C_{i}^{A}=C^{T}\times \omega \left(CON_{i}\right),\;\mathrm{where}\;0\leq \omega \left(CON_{i}\right)\leq 1. \end{array}$$

The fundamental principle of our proportional share scheduling is similar to max–min-like policies, because the credit-based algorithm is a computationally efficient substitute for them. Both algorithms can distribute network resources in proportion to the weight, but credit-based scheduling is much simpler and more computationally efficient [32].

#### 3.3.2. Support for Work-Conserving

qCon supports a work-conserving policy [31] to improve network resource utilization. Work-conserving keeps network resources busy by allowing a virtual network interface of a container with no requests to yield its allocated network resources to other virtual network interfaces, which are ready to be scheduled. Work-conserving is an important factor in fog computing, as it allows network resource utilization to be maximized. For this purpose, when container $CON_{i}$ does not fully consume its allocated credit during the current scheduling period, the remaining credit is added to the total credits of the next scheduling period and distributed to other containers. $C_{i}^{R}$ indicates the remaining credit of container $CON_{i}$ at the current scheduling period. This credit amount is distributed to other containers in proportion to their weights.

When we assume that container $CON_{j}$ receives the remaining credit from container $CON_{i}$, we first obtain the fair share of container $CON_{j}$, $C_{j}^{A}$, at the next scheduling period as follows:

(2) $$\begin{array}{cc} C_{j}^{A}= & C^{T}\times \omega \left(CON_{j}\right)+C_{i}^{R}\times \frac{1}{1-\omega (CON_{i})}\times \omega \left(CON_{j}\right), \end{array}$$

(3) $$\begin{array}{cc} & =\left(C^{T}+C_{i}^{R}\times \frac{1}{1-\omega (CON_{i})}\right)\times \omega \left(CON_{j}\right). \end{array}$$

Next, we generalize this equation. We assume that container $CON_{j}$ receives the remaining credits from other containers, yielding their allocated network resources as well. We then obtain the fair share of container $CON_{j}$, $C_{j}^{A}$, at a certain scheduling period as follows:

(4) $$\begin{array}{ccc} C_{j}^{A} & = & C^{T}\times \omega \left(CON_{j}\right)+\sum_{i=1}^{n}\left(C_{n}^{R}\times \frac{1}{1-\omega (CON_{n})}\right)\times \omega \left(CON_{j}\right). \end{array}$$

According to Equation (4), every container receives a greater credit amount than its fair share amount calculated by Equation (1), which makes the sum of credits of all containers greater than $C^{T}$. We correct this situation by deducting credits from containers that yield their allocated network resources. This deduction is reasonable because such containers tend to underutilize network resources for several scheduling periods. When $C_{l}^{AT}$ indicates a temporary credit value of container $CON_{l}$ received by Equation (4), we obtain the fair share of container $CON_{l}$ that yields its allocated network resources to other containers at a certain scheduling period as follows:

(5) $$\begin{array}{ccc} C_{l}^{A} & = & C_{l}^{AT}-C_{l}^{R}. \end{array}$$

#### 3.3.3. Minimum Bandwidth Reservation and Maximum Bandwidth Limitation

Minimum bandwidth reservation and maximum bandwidth limitation are performance policies to ensure a quantitative performance guarantee in controlling the network performance of containers. When the minimum bandwidth reservation policy is applied to container $CON_{i}$, qCon ensures that the network performance of container $CON_{i}$ is greater than the configured value, $C_{i,min}^{A}$. Container $CON_{i}$ receives a credit of $C_{i}^{A}$ by proportional share scheduling with work-conserving (Section 3.3.1 and Section 3.3.2) at every scheduling period. When this fair share value, $C_{i}^{A}$, is below the configured value, $C_{i,min}^{A}$, minimum bandwidth reservation changes the value of $C_{i}^{A}$ to $C_{i,min}^{A}$ to ensure minimum bandwidth while retrieving network resources from other containers in proportion to their weights. Obviously, the aggregated amounts of minimum bandwidth requests from all containers should not exceed the total amount of credits, $C^{T}$.

When we assume that the fair share value, $C_{i}^{A}$, is below the configured value, $C_{i,min}^{A}$, the received credit amount of container $CON_{i}$ by minimum bandwidth reservation at a certain scheduling period is as follows:

(6) $$\begin{array}{ccc} C_{i}^{A} & = & C_{i,min}^{A}. \end{array}$$

At this time, the network resources of other containers are retrieved. When *k* is the set of containers having minimum bandwidth reservation, we obtain the credit amount of container $CON_{j}$ whose network resources are retrieved, $C_{j}^{A}$, at a certain scheduling period as follows:

(7) $$\begin{array}{ccc} C_{j}^{A} & = & C^{T}\times \omega \left(CON_{j}\right)-\sum_{k}\left(\left(C_{k,min}^{A}-C_{k}^{A}\right)\times \frac{1}{1-\omega (CON_{k})}\right)\times \omega \left(CON_{j}\right). \end{array}$$

To prevent the case where the sum of the minimum bandwidths of applications exceeds the total bandwidth, $C^{T}$, qCon applies an admission control policy. qCon rejects further minimum bandwidth reservation requests when it does not have sufficient available resources. Therefore, qCon can satisfy existing containers’ minimum bandwidth reservations without request crashes. The container that issued the rejected request can be migrated to another fog node that has enough network resources by an orchestration mechanism [33]. In future work, we plan to adopt an orchestration mechanism to automatically migrate containers with rejected requests.

Maximum bandwidth limitation prevents containers from exceeding their performance limits, which is achieved by redistribution of surplus credits of containers that have maximum bandwidth limitation. When the maximum bandwidth limitation policy is applied to container $CON_{i}$, qCon enforces that the network performance of container $CON_{i}$ is not greater than the configured value, $C_{i,max}^{A}$. When the fair share value by proportional share scheduling, $C_{i}^{A}$, is above the configured value, $C_{i,max}^{A}$, maximum bandwidth limitation changes the value of $C_{i}^{A}$ to $C_{i,max}^{A}$ to enforce maximum bandwidth while re-distributing surplus network resources from container $CON_{i}$ to other containers in proportion to their weights.

When we assume that the fair share value, $C_{i}^{A}$, is above the configured value, $C_{i,max}^{A}$, the received credit amount of container $CON_{i}$ by maximum bandwidth limitation at a certain scheduling period is as follows:

(8) $$\begin{array}{ccc} C_{i}^{A} & = & C_{i,max}^{A}. \end{array}$$

At this time, the surplus network resources of container $C_{i}^{A}$ are distributed to other containers. When *k* is the set of containers having maximum bandwidth limitation, we obtain the credit amount of container $CON_{j}$ that receives surplus network resources, $C_{j}^{A}$, at a certain scheduling period as follows:

(9) $$\begin{array}{ccc} C_{j}^{A} & = & C^{T}\times \omega \left(CON_{j}\right)+\sum_{k}\left(\left(C_{k}^{A}-C_{k,max}^{A}\right)\times \frac{1}{1-\omega (CON_{i})}\right)\times \omega \left(CON_{j}\right). \end{array}$$

#### 3.3.4. Combination of the Three Scheduling Policies

Algorithm 1 shows the credit calculation function of qCon, where the three scheduling policies (i.e., proportional share scheduling, minimum bandwidth reservation, and maximum bandwidth limitation) are combined with the work-conserving mechanism. This function performs iteration from the first container to the last one to calculate each container’s credit value at the current scheduling period according to the performance requirements. In the algorithm, *total_weight* denotes the sum of the weights of all containers. *weight_left* is initialized as *total_weight*, and indicates the sum of the weights of remaining containers during the *for* loop. *total_credit* is the total amount of credits that will be distributed to all containers. *credit_left* is initialized as zero, and is a temporary variable to save the remaining credit when a container does not fully consume its allocated credit.

**Algorithm 1:** Combination of the three scheduling policies with the work-conserving mechanism. total_weight ← the sum of weights of all containers; weight_left ← total_weight; total_credit ← the total amount of credits; credit_left ← 0; 
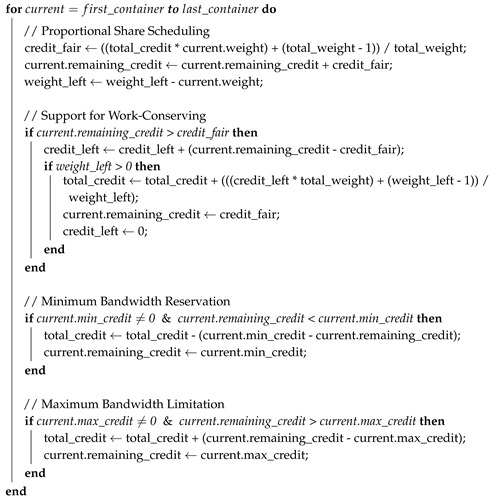


At each iteration of the *for* loop, *current* denotes the current container to be processed. The first part of the algorithm applies proportional share scheduling. It obtains the current container’s fair share credit value, *credit_fair*, based on two factors: the total amount of credits and the container’s weight. Then, it adds the obtained value to the remaining credit value of the current container (i.e., *remaining_credit*). The remaining credit value is the cumulative credit amount. The second part performs work-conserving. When the current container’s remaining credit value is greater than the fair share amount, the unused credit value in the previous scheduling period is added to *total_credit* so that it can be distributed to other containers. This procedure is performed if there are some remaining containers to be processed in the next iterations (i.e., *weight_left* > 0). The third part is for processing minimum bandwidth reservation. When the current container is configured to reserve minimum bandwidth and at the same time lacks sufficient resources, the container retrieves network resources from other containers. The final part processes maximum bandwidth limitation. When the current container is configured to limit maximum bandwidth, the container re-distributes surplus network resources to other containers.

## 4. Evaluation

**Figure 6:**
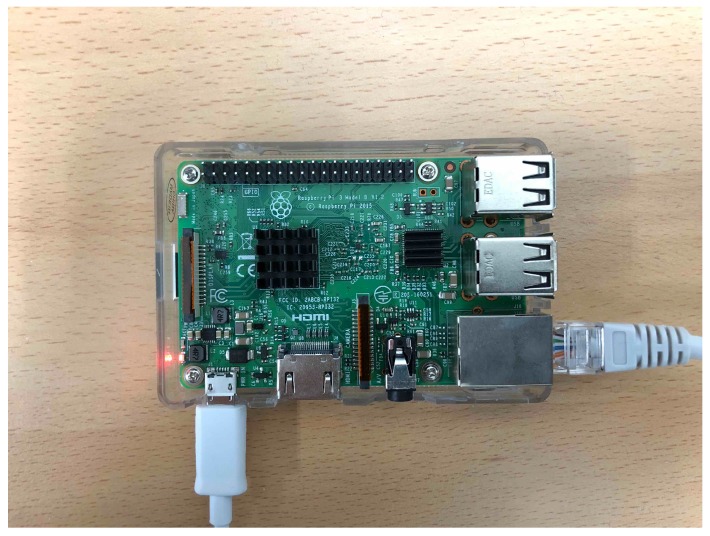
Raspberry Pi 3 Model B used in the evaluation.

We present the performance evaluation results of qCon in this section. We implemented qCon on a Raspberry Pi 3 Model B board as shown in Figure 6, which had an ARMv7 64-bit quad core CPU running at 1.2 GHz, 1 GB of RAM, and a 100 Megabit Ethernet chip. We used Ubuntu Linux 16.04 for the Raspberry Pi board, and its kernel version was v4.4.38. The Raspberry Pi board is a small single-board computer that runs multiple containers managed by Docker with version 18.03. We used another x86 server for receiving packets from the Raspberry Pi board. The server had an Intel i7-3930K Hexa core CPU running at 3.2 GHz, 16 GB of RAM, and a Gigabit Ethernet card. As the Raspberry Pi card has a 100 Megabit Ethernet chip, the connection speed between the two machines was limited to 100 Mbps. In our evaluation, each container ran a client of a network benchmark program called netperf, which transmitted 16 KB TCP packets for 60 s. The x86 server ran a netperf server that received the transmitted packets from the client and measured the network throughput.

### 4.1. Multiple Scheduling Policies

qCon supports the following three scheduling policies to satisfy various performance requirements of containers in fog computing: proportional share scheduling, minimum bandwidth reservation, and maximum bandwidth limitation. We first applied each scheduling policy respectively to containers and observed the network performance of each container. We then applied the three scheduling policies together to containers.

#### 4.1.1. Proportional Share Scheduling Evaluation

First, we evaluated proportional share scheduling when various weight values were assigned to three containers C1, C2, and C3 running concurrently on the Raspberry Pi board. We applied three sets of weight values to the three containers for three experiments. The weight sets were as follows:

- Weight set *1 to 1* : {1, 1, 1};
- Weight set *1 to 3* : {1, 2, 3};
- Weight set *1 to 5* : {1, 3, 5}.

Each weight set represents the target performance ratio for containers C1, C2, and C3. For example, weight set *1 to 5* means that the ratio of network performance would converge to 1:3:5 for containers C1, C2, and C3. If the experiment results met the target ratio, we could conclude that qCon successfully differentiated the network performance of containers based on the weight values.

**Figure 7:**
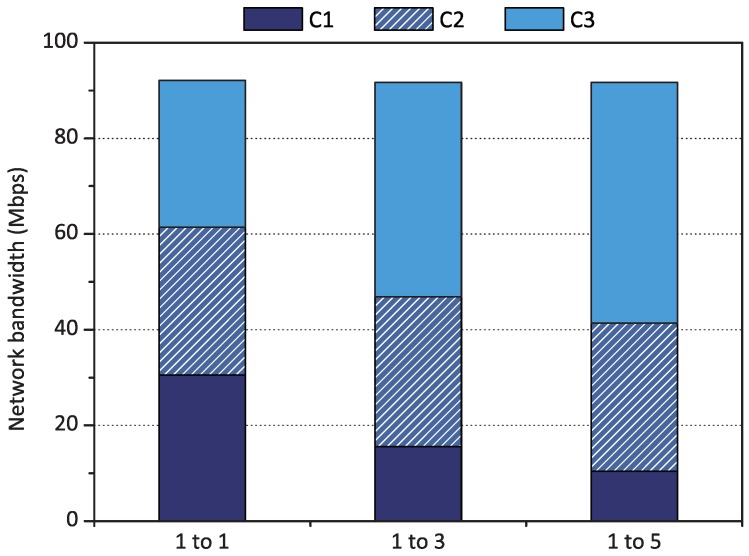
Proportional share scheduling evaluation results of qCon when the weight sets were *1 to 1*, *1 to 3*, and *1 to 5*.

Figure 7 shows that qCon effectively differentiated the network performance of each container depending on each weight set. As qCon allocates credits to containers according to their weights, the network performance of each container was proportionally differentiated, as expected. When weight set *1 to 1* was applied, three containers had the same bandwidth. When weight set *1 to 3* was configured, container C3 achieved 43 Mbps of bandwidth while container C1 showed reduced bandwidth, 16 Mbps, compared to weight set *1 to 1*. When we assigned weight set *1 to 5*, the network performance of container C3 increased by five times compared to container C1.

**Figure 8:**
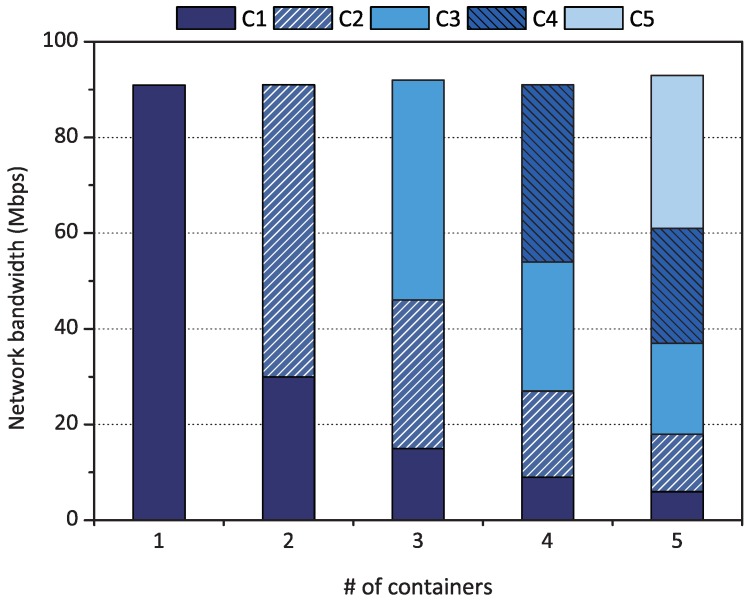
Proportional share scheduling evaluation results of qCon when the number of containers running concurrently increased from one to five (weight set was *1 to 5*).

In addition, we measured the network performance of containers when the number of containers running concurrently increased from one to five. We utilized the weight set *1 to 5* for the containers, which means that the ratio of network performance would converge to 1:2:3:4:5 for containers C1, C2, C3, C4, and C5. As depicted in Figure 8, when C1 executed netperf alone, C1 utilized the entire network bandwidth of the Raspberry Pi board. As explained in Section 3.3.2, qCon supports a work-conserving policy that allows a container to receive more credits than its fair share when there are unused network resources. As containers C2, C3, C4, and C5 started to run one-by-one, the network performance of each container was differentiated in proportion to its weight. For example, when the five containers ran simultaneously, container C4 achieved 24 Mbps, which was twice the performance of container C2 (12 Mbps).

#### 4.1.2. Minimum Bandwidth Reservation Evaluation

**Figure 9:**
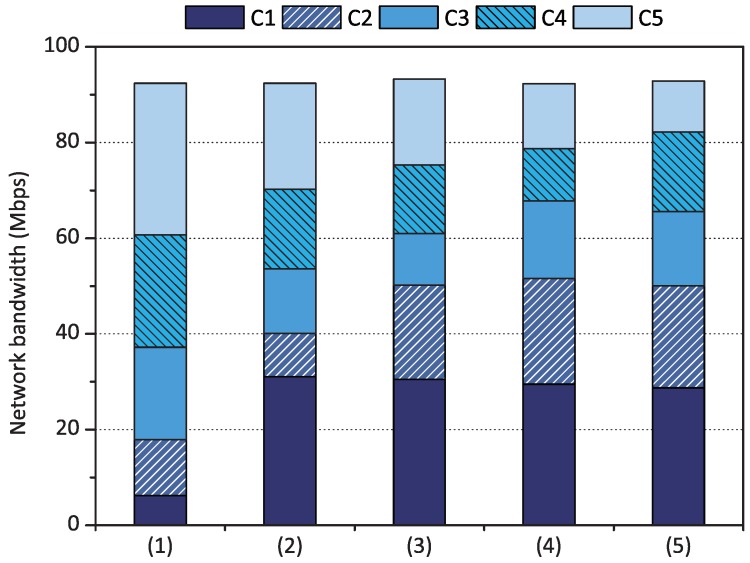
Minimum bandwidth reservation results of qCon when (1) proportional share scheduling was applied with weight set *1 to 5*, (2) minimum bandwidth was configured as [C1 = 30 Mbps] with weight set *1 to 5*, (3) [C1 = 30 Mbps, C2 = 20 Mbps] with weight set *1 to 5*, (4) [C1 = 30 Mbps, C2 = 20 Mbps, C3 = 15 Mbps] with weight set *1 to 5*, and (5) [C1 = 30 Mbps, C2 = 20 Mbps, C3 and C4 = 15 Mbps] with weight set *1 to 5*.

Then, we applied the minimum bandwidth reservation policy while proportional share scheduling with weight set *1 to 5* was applied to each container. The first case of Figure 9 shows the result when proportional share scheduling was only applied to each container. Then, we assigned 30 Mbps to container C1 as its minimum bandwidth. As shown in the second case of Figure 9, the performance of C1 increased by up to 31 Mbps, which satisfied the minimum bandwidth condition. As qCon retrieves network resources from other containers as explained in Equation (7) of Section 3.3.3, the performance of containers C2, C3, C4, and C5 decreased compared to the first case. Then, we assigned 20 Mbps to container C2 as its minimum bandwidth while maintaining the proportional share scheduling condition and container C1’s minimum bandwidth. Similarly, the performance of container C2 increased by up to 20 Mbps while preserving container C1’s minimum bandwidth reservation, as shown in the third case of Figure 9. In the fourth case, we assigned 15 Mbps to container C3, and its minimum bandwidth could be reserved. In the last case, we configured each minimum bandwidth of containers C1, C2, C3, and C4 as 30, 20, 15, and 15 Mbps, respectively. As shown in the last case, the containers with minimum bandwidth reservation could achieve the configured bandwidth. Container C5 without minimum bandwidth reservation showed decreased network performance as it received the remaining network resources after other containers’ minimum bandwidth reservation was completed.

#### 4.1.3. Maximum Bandwidth Limitation Evaluation

**Figure 10:**
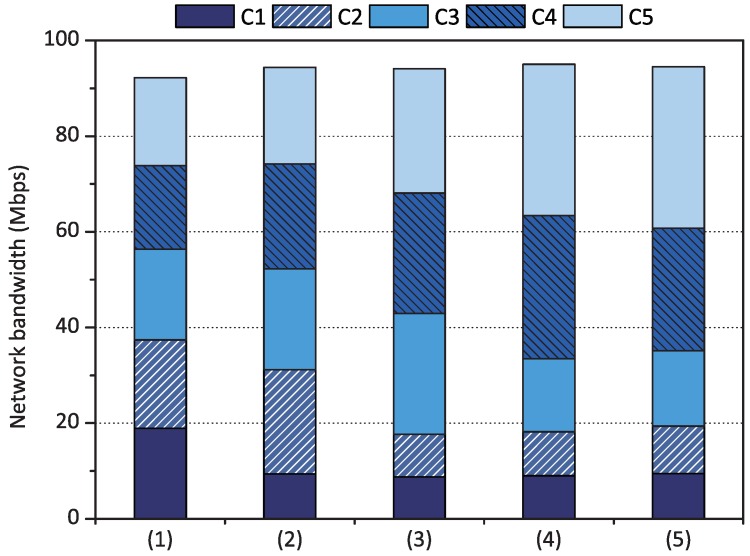
Maximum bandwidth limitation results of qCon when (1) proportional share scheduling was applied with weight set *1 to 1*, (2) maximum bandwidth limitation was set to [C1 = 10 Mbps] with weight set *1 to 1*, (3) [C1 and C2 = 10 Mbps] with weight set *1 to 1*, (4) [C1 and C2 = 10 Mbps, C3 = 15 Mbps] with weight set *1 to 1*, and (5) [C1 and C2 = 10 Mbps, C3 = 15 Mbps, C4 = 25 Mbps] with weight set *1 to 1*.

Next, we evaluated the maximum bandwidth limitation policy when proportional share scheduling with weight set *1 to 1* was applied to each container. The first case of Figure 10 shows the result of proportional share scheduling. Then, we assumed that container C1 had used the quotas allowed for a day, and bandwidth limitation needed to be enforced on the container. We assigned 10 Mbps to container C1 as the maximum bandwidth limitation. In this case, qCon retrieved the container’s excessive credit and re-distributed it to other containers as explained in Equation (9) of Section 3.3.3. Therefore, the network performance of C1 decreased by up to 9 Mbps while an amount of 9 Mbps was re-distributed to other containers in proportion to their weights, as shown in the second case of Figure 10. The third case shows the performance result when we assigned 10 Mbps to containers C1 and C2, respectively, as maximum bandwidth limitation. Then, we limited the performance of containers C3 and C4 to 15 and 25 Mbps, respectively, as shown in the fourth and last cases of Figure 10. In the last case, the performance of containers C3 and C4 decreased by up to 15 and 25 Mbps while qCon preserved the maximum bandwidth limitations of containers C1 and C2 and increased the performance of C5 by up to 34 Mbps.

#### 4.1.4. Evaluation of Concurrent Support of Multiple Policies

**Figure 11:**
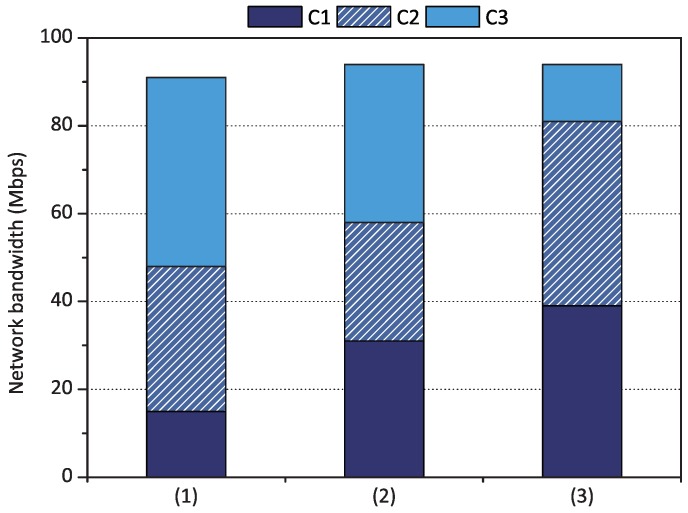
Network performance of the three scheduling policies when (1) proportional share scheduling was applied with weight set *1 to 3*, (2) container C1’s minimum bandwidth reservation was set to 30 Mbps with weight set *1 to 3*, and (3) container C3’s maximum bandwidth limitation was set to 15 Mbps with weight set *1 to 3* and C2’s minimum bandwidth reservation.

Finally, the three scheduling policies of qCon were applied simultaneously to a fog device. qCon adjusts the performance of containers when the obtained performance calculated by proportional share scheduling does not meet the minimum reservation or maximum limitation performance. This is because minimum bandwidth reservation and maximum bandwidth limitation have a higher priority than proportional share scheduling. Figure 11 shows the performance results of each container when the scheduling policies were applied cumulatively. The first case of the figure shows the result when proportional share scheduling was only applied to each container with weight set *1 to 3*. When we applied minimum bandwidth reservation for container C1 to achieve 30 Mbps, the performance of C1 increased by up to 31 Mbps while the performance of C2 and C3 decreased as shown in the second case of the figure. Finally, when we limited the performance of container C3 to 15 Mbps, the surplus resources of container C3 were re-distributed to containers C1 and C2 depending on their weights, which increased the performance of containers C1 and C2 as shown in the last case of the figure.

### 4.2. CPU Overhead

In fog computing, it is important to decrease CPU overhead in order to achieve energy efficiency, as up-to-date fog technologies employ energy-limited devices. We evaluated the CPU overhead caused by qCon in terms of CPU utilization. We also compared the results with native Docker and Linux Traffic Control, which controls the network performance of containers similar to qCon. In Linux Traffic Control and qCon, we applied proportional share scheduling and configured all containers to have the same weight.

**Figure 12:**
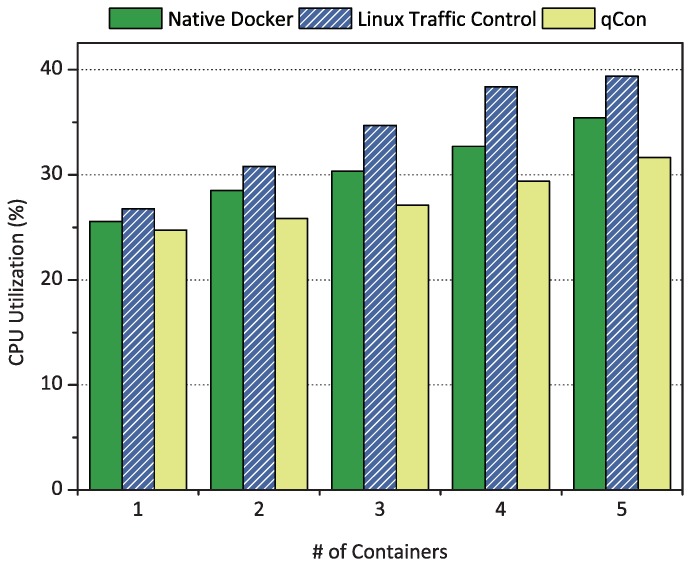
CPU utilization of the target board with the increase of active containers from one to five.

Figure 12 shows the CPU utilization of the target board when the number of containers running the netperf client increased from one to five consecutively. In this figure, qCon achieved the lowest CPU overhead compared to native Docker and Linux Traffic Control. qCon reduced the CPU utilization of the target board by elaborately controlling the network resource consumption of each container with the new scheduling algorithm. This enabled efficient packet processing in the Linux bridge by eliminating resource contention between co-located containers. On the other hand, Linux Traffic Control showed the highest CPU utilization because of its *spinlock* implementation. Linux Traffic Control utilizes spinlocks for synchronization between multiple cores. When the number of containers increases, the number of cores for processing packets is also increased, which grows synchronization bottlenecks [34].

### 4.3. Network Latency

In this section, we measured the network latency of qCon and compared the results with native Docker and Linux Traffic Control using the same experimental setup explained in Section 4.2. We used a *ping* program that sent 64-byte UDP packets to the evaluation server to measure the latency. Table 1 demonstrates that qCon did not incur additional latency compared to native Docker. This is because qCon does not intervene in packet processing in the Linux bridge, but limits the transmission rate of each container according to the configured scheduling policy. The Linux Traffic Control also showed similar latency to qCon.

## 5. Related Work

In centralized cloud computing, a number of techniques have been proposed to enable virtualized devices to support QoS [13,35,36,37,38]. In particular, network interfaces are regarded as challenging devices for ensuring QoS, because many scheduling layers are involved in packet processing in virtualization.

Lee et al. [36] proposed a network virtualization framework that differentiates the network performance of VMs by allocating network resources in proportion to the CPU usage. The framework is based on a leaky bucket controller with a time slot-based resource allocator. DMVL [38] provides a technique to assign certain network bandwidth to each VM in a fair-share manner. For this purpose, it offers different I/O queues to each virtual machine and separates the logical data path for packet processing. It also monitors the network resource usage of each VM by using shared memory between the driver domain and the VM, which is used for the adjustment of resource allocation.

Most of the techniques for VMs process packets based on a rate-limiting approach [39], which controls the transmission rate of each VM. However, this approach incurs non-negligible CPU overhead for network performance management [40]. Rate limiting generally needs to analyze and classify packets from VMs based on their source or destination addresses, and this requires an amount of computation resources. This situation makes it difficult to adopt rate limiting in container-based fog computing, as fog computing often employs performance-limited devices.

ANCS [13] is our previous work and achieves QoS in Xen-based VMs. Compared to the rate-limiting approach, ANCS incurs lower overhead by adopting credit-based scheduling, as with qCon. However, ANCS focuses on a virtual machine (VM) environment in a data center where computation and network resources are redundant. ANCS runs in an additional kernel thread in the Xen hypervisor to schedule network resources. The kernel thread fetches network requests from the backend driver in the driver domain (i.e., domain0) of Xen and sends packets according to its scheduling policy. However, the kernel thread requires a dedicated processor core for preventing packet delays in the backend driver. Different from ANCS, qCon targets fog computing, which means qCon needs to be redesigned much lighter than ANCS. In order to minimize computation resources for network resource management, qCon eliminates the kernel thread of ANCS. Instead, qCon is embedded in the Linux bridge and conducts network scheduling during packet processing of the Linux bridge in a synchronized manner. This new design does not require a dedicated processor core and is suitable for performance-limited fog devices, which have smaller cores.

Network resource management for containers can be enabled by using *cgroups* and Linux Traffic Control. *cgroups* assign the specific bounds of resource usage to a container, and Linux Traffic Control processes each container’s packets according to the configured scheduling policy. However, *cgroups* and Linux Traffic Control only provide limited functionalities, which cannot support the efficient control of network bandwidth for QoS management, as explained in Section 1. For example, the existing scheduling policies of Linux Traffic Control such as the Deficit Round-Robin (DRR) scheduler [23] and the Hierarchical Token Bucket (HTB) queuing discipline [11] do not offer complete proportional sharing or minimum reservation. Dusia et al. [12] utilized Linux Traffic Control to enable priority-based network scheduling for Docker containers. Compared to these studies, qCon enables a proportional share scheduling policy, which is essential for fog computing to give access to network resources on a pay-per-use basis. qCon can also apply multiple performance policies, including proportional share scheduling, minimum reservation, and maximum limitation to containers concurrently.

Most studies on network resource management for containers mainly focus on developing QoS-aware orchestration systems [33,41,42,43]. In fog and edge computing, devices have limited network capabilities, and this limitation causes the fog and edge devices to only perform until they reach the maximum network capacity. QoS-aware orchestration systems exploit several fog and edge nodes to load-balance workloads or deploy an application in an appropriate node while considering the level of QoS. Brogi et al. [41] proposed a general model for the QoS-aware deployment of IoT applications. The model determines where to deploy an IoT application in a fog infrastructure through recursive searching. Similarly, Pavsvcinski et al. [33] presented an orchestration technique that places network-intensive utilities while considering the geographical information of the fog nodes. They implemented the proposed technique in an open source container system called Kubernetes, and showed that their solution was able to find the most appropriate fog node to deploy the network utilities. Skarlat et al. [42] presented the fog service placement problem (FSPP), which determines the placement of IoT services on virtualized fog resources with a consideration of QoS constraints. The FSPP aims to discover optimal mapping between IoT services and the fog resources, which satisfies the QoS requirements and achieves high resource utilization.

qCon is complementary to these orchestration systems. Even with a QoS-aware orchestration tool, performance interference can occur between co-located containers running on the same machine. The co-located containers share the same computing resources (e.g., the CPU, memory, and network devices). When a specific container consumes the network resources aggressively, other containers on the same node may experience resource starvation, which cannot guarantee the configured QoS by the QoS-aware orchestration system [43]. By offering both proportional fair scheduling and bandwidth shaping, qCon enables the enforcement of the resource allocation determined by the orchestration system.

## 6. Discussion

In the open internet, bandwidth cannot be reserved without help of intermediate routers. Therefore, we expect that qCon can be employed in a network environment where qCon can send traffic with qCon’s scheduling policies, and a neighbor node located within a single-hop distance receives the traffic according to the controlled bandwidth. As explained in the use cases in Section 1, qCon can be employed by IoT gateways in a smart factory, and the IoT gateway can send traffic received from sensors to a neighbor server in the same factory for analyzing the data. In addition, a device employing qCon can be utilized as a fog node where multiple tenants directly connect to the device. In this use case, qCon controls outbound traffic to the tenants according to the performance policy. In these scenarios, the scheduling policies of qCon can be reserved as qCon’s device and its neighbor nodes are within a single-hop distance.

As with other traffic control mechanisms [12,44], qCon performs buffering on both TCP and UDP traffic. An effect of buffering of UDP packets is to limit the rate of outbound traffic by delaying the sending of UDP packets. For example, suppose that a container generates a very high volume of UDP traffic at one burst. Then, other containers sending TCP traffic would be influenced by the massive UDP packets, resulting in low bandwidth and high latency. qCon’s scheduling can prevent this situation with proportional share scheduling by controlling the container with UDP traffic to send traffic at a stable rate according to its weight.

qCon focuses on outgoing traffic for bandwidth control, as with other traffic control mechanisms [12,44]. In principle, it is difficult to limit the rate of inbound traffic without any cooperation from outside network devices [45]. Therefore, qCon does not perform bandwidth control on inbound traffic. As explained in the use cases in Section 1, qCon limits the rate of outbound TCP and UDP traffic to satisfy the various performance requirements of containers.

## 7. Conclusions

In this paper, we proposed qCon, which is a QoS-aware network resource management framework for containers in fog computing for IoT. In a container environment, qCon can provide both proportional 16 share scheduling and bandwidth shaping to containers in order to meet various performance demands. qCon also implements its own scheduling framework on the Linux bridge, which incurs low CPU overhead.

References

## Figures and Tables

**Table 1:** Network latency (ms) measured using ping under native Docker, Linux Traffic Control, and qCon.

Platform	Native Docker	Linux Traffic Control	qCon
Latency (ms)	0.5	0.6	0.5
